# Coach-Created Motivational Climate and Athletes’ Adaptation to Psychological Stress: Temporal Motivation-Emotion Interplay

**DOI:** 10.3389/fpsyg.2019.00617

**Published:** 2019-03-22

**Authors:** Montse C. Ruiz, Claudio Robazza, Asko Tolvanen, Saara Haapanen, Joan L. Duda

**Affiliations:** ^1^Faculty of Sport and Health Sciences, University of Jyväskylä, Jyväskylä, Finland; ^2^BIND-Behavioral Imaging and Neural Dynamics Center, Department of Medicine and Aging Sciences, Università degli Studi G. d’Annunzio Chieti e Pescara, Chieti, Italy; ^3^Faculty of Education and Psychology, University of Jyväskylä, Jyväskylä, Finland; ^4^School of Sport, Exercise and Rehabilitation Sciences, University of Birmingham, Birmingham, United Kingdom

**Keywords:** feelings, psychobiosocial states, IZOF model, achievement goal theory, self-determination theory, structural equation modeling

## Abstract

This two-wave study investigated the temporal interplay between motivation and the intensity and reported impact of athletes’ emotions in training settings. In total, 217 athletes completed self-report measures of motivational climate, motivation regulations, emotional states (i.e., pleasant states, anger, and anxiety) experienced before practice at two time points during a 3-month period. Latent change score modeling revealed significantly negative paths from task-involving climate at time 1 to the latent change in the intensity of dysfunctional anxiety and anger, and significantly positive paths from ego-involving climate at time 1 to the latent change in dysfunctional anger (i.e., intensity and reported impact). The paths from controlled motivation at time 1 to the latent change in the intensity of dysfunctional anxiety and vice versa were significantly positive. The path from controlled motivation at time 1 to the latent change in the intensity of functional anger was significantly positive, but not vice versa. In addition, the paths from dysfunctional anger (i.e., intensity and reported impact) at time 1 to the latent change in motivation regulations were significant, but not vice versa. Overall, evidence provided suggested that the temporal interplay of motivation and emotions is contingent on the specific emotions. The findings highlight the role of coach-created motivational climate and the importance of identifying high levels of controlled motivation to help athletes better adapt to psychological stress.

## Introduction

The focus of existing sport emotion literature has been on the prediction of performance (Beedie et al., 2000; Woodman et al., 2009) or the strategies athletes use to regulate their emotions in order to enhance performance (Lane et al., 2012; Wagstaff, 2014). The antecedents of performance related emotions in sport, however, have received less research attention. The purpose of this study was to examine the social environmental antecedents of and the interplay between emotions and motivation. Understanding these antecedents can provide useful information to coaches and practitioners to help athletes enhance their adaptation to psychological stress related to their performance in high achievement settings.

Theorists and research evidence suggest that the social environment and individual variables influence the way people think, feel, and behave (Nicholls, 1989; Deci and Ryan, 2000; Lazarus, 2000). Two prominent theoretical frameworks used in the study of motivation are achievement goal theory (AGT; Nicholls, 1989), and self-determination theory (SDT; Deci and Ryan, 1985, 2000; Ryan and Deci, 2017). These theories have been applied to examine the intrapersonal motivational and emotional consequences of the social environment in the sporting context.

According to AGT, a task-involving climate is defined by situations where the coach focuses on skill improvement, individual progress, and encourages cooperation with others, and in which every individual has an important role in the team. In contrast, an ego-involving climate involves the use of normative-based evaluation, emphasis on competition, and social comparison between participants. In line with SDT, individuals’ motivation varies in their degree of self-determination. For example athletes experience autonomous motivation when their reasons for engagement in sport are volitional or intrinsic, while controlled motivation is experienced when the reasons for engagement are pressured either internally or externally. These reasons for engagement lie on a continuum from intrinsic to extrinsic motivation. The most autonomous form of motivation is intrinsic motivation, which occurs when athletes derive a sense of enjoyment and satisfaction from participating in sport. In contrast, extrinsic motivation involves participation that is contingent upon specific reward or outcomes. For instance, integrated regulation, which is the most autonomous form of extrinsic motivation, occurs when athletes view participation in sport as personally important and assimilated with their own self. Identified regulation occurs when the outcome of a sport is personally valued. Introjected regulation is reflected when athletes engage in a sport to reduce feelings of shame or guilt. The most controlled form of motivation is external regulation, which is manifested when athletes engage in an activity for purely instrumental reasons, such as obtaining reward or satisfying an external demand, while a lack in motivation has been referred to as amotivation (Deci and Ryan, 1985; Ryan and Deci, 2000, 2017). Researchers have typically examined autonomous motivation as comprised by intrinsic motivation, integrated regulation, and identified regulation, while introjected regulation and external regulation were indicators of controlled motivation (Lonsdale et al., 2008; Langan et al., 2016).

The vast majority of research in this area has placed motivational climate as the antecedent of intrapersonal variables. However, the examination of the temporal sequence of intrapersonal variables, such as athletes’ motivation and emotions as predictors or determinants, remains unexplored. According to both AGT and SDT, much of the variance in individuals’ motivation and quality of involvement derives from the interaction with significant others, such as the coaches within sport contexts. Research evidence indicates that perceptions of a task-involving climate are related to a more functional/adaptive motivational pattern, intrinsic motivation, and achievement striving in sport, while the opposite has been found for an ego-involving climate (for review, see Harwood et al., 2015). Overall, research has generally revealed that a task-involving climate is related to athletes’ intrinsic motivation and need satisfaction (Reinboth and Duda, 2006; Vazou et al., 2006; Álvarez et al., 2012; for an overview see Duda and Balaguer, 2007). A task-involving climate has also been found to be a positive predictor of more self-determined styles of motivation (Standage et al., 2003; Kipp and Amorose, 2008). In contrast, an ego-involving climate has been related to feelings of pressure, antisocial behavior, the belief of ability as determinant of success, and dropping out in sport (Sarrazin et al., 2002; Bortoli et al., 2012). This maladaptive motivational pattern reflects a lack of adaptation to psychological stress. An ego-involving climate was also found to positively predict extrinsic motivation and amotivation (Bortoli et al., 2015; Jaakkola et al., 2017).

Regarding the relationships between the social environment created by coaches and athletes’ emotional responses, research has shown that perceptions of a task-involving motivational climate significantly predicted pleasant states in soccer players (Bortoli et al., 2012) and enjoyment in young hockey players (Jaakkola et al., 2016). In contrast, perceptions of an ego-involving climate were predictors of unpleasant states, anxiety, worry, and decreased enjoyment (Vazou et al., 2006; Cumming et al., 2007; Bortoli et al., 2012). A systematic review of 39 studies (Harwood et al., 2015) indicates a moderate positive correlation between perceived task-involving motivational climate and pleasant affect, while an overall small, negative correlation was found between an ego-involving motivational climate and pleasant states.

The vast majority of research exploring the relationships between motivational climate, motivation, and emotions is cross-sectional in nature. Thus, the direction of causality in these relationships remains unexamined. Previous research in this area has examined the consequences of the social motivational context investigating two possible sequences. The first sequence considers that motivational climate dimensions serve as antecedents of variability in motivation regulations, which in turn, trigger different emotions. The second sequence assumes that emotion is a mediator in the relationship, and thus, motivation is positioned at the end of the sequence. AGT and SDT postulate that the social environment and achievement goals have emotional, cognitive, and behavioral consequences (Deci and Ryan, 1985; Nicholls, 1989; Ames, 1992; Duda and Balaguer, 2007; Ryan and Deci, 2017). Motivational climate and motivation (especially a task-involving climate, and autonomous motivation) are assumed to influence performance with emotion mediating this relationship. Also in line with the first sequence (motivational climate > motivation > emotion), Lazarus (2000) placed importance on causal cognitive, motivational, and relational aspects in the initiation and maintenance of emotions. He stated that individuals’ emotions result from appraisals about the personal significance of the interaction with others and the environment, and options for coping with situational demands. Emotions would, thus, be placed at the end of the sequence. The second sequence (motivational climate > emotion > motivation) has been mainly tested within physical education settings and youth sport (Bortoli et al., 2009, 2011, 2012, 2014). The main focus in such contexts is typically on the creation of an environment that would enhance pleasant states (e.g., enjoyment), which are believed to increase the motivation to be involved in the activity.

Both sequences, however, have been examined separately. Moreover, most previous studies have involved school or college participants, with a few studies recruiting high-performing athletes, young athletes in particular. In addition, the vast majority of studies have been limited to the examination of one dimension of emotions, intensity. Another important dimension in the sporting context is the functional impact on performance. A very few studies have considered this dimension, which has been for the most part only assumed by researchers. For example, Bortoli et al. (2014) implied pleasant states as functional states and unpleasant states as dysfunctional; however, athletes’ perceptions of the functional impact on performance were not assessed.

Traditionally, athletes’ emotions have been studied using either a global affect approach, which emphasizes dimensions such as hedonic tone and activation (Watson and Tellegen, 1985), or a discrete emotion approach, which considers emotion as distinct entities (e.g., anxiety, anger) triggered by the person’s appraisal of their interaction with the environment (Lazarus, 2000). One sport-specific theoretical framework concerned with the study of emotions is the individual zones of optimal functioning (IZOF) model (Hanin, 2000, 2007). The IZOF model, which combines global affect and discrete emotion perspectives, conceptualizes emotions within the framework of two interrelated factors, hedonic tone (i.e., pleasure–displeasure), and performance functionality (i.e., functional-dysfunctional effects). This categorization results in a range of pleasant and unpleasant, functional and dysfunctional emotional experiences. Extensive empirical evidence supports this conceptualization (for reviews, see Ruiz et al., 2017b; Robazza and Ruiz, 2018). Most IZOF-based research, however, has focused on the emotion-performance relationship disregarding the study of the antecedents of emotional states.

A study examining the interplay between motivational climate, motivation, and the intensity and functional impact of athletes’ emotions revealed that task-involving climate was a positive predictor of autonomous motivation and perceived functional anger, and a negative predictor of the intensity of anxiety and dysfunctional anger (Ruiz et al., 2017a). An ego-involving climate was a positive predictor of controlled motivation, the intensity and perceived impact of functional anger, and the intensity of dysfunctional anger. Such study involved data assessed at one moment in time, which did not allow for the examination of the mediating role of motivation versus emotions in the motivational climate–outcome relationship.

### Current Study and Hypotheses

To our knowledge, no study has yet examined the relationship between pleasant and stress-related (i.e., anxiety and anger) emotions and motivation over time prior to practice. The time-lagged design of the present study allows addressing this gap in the literature, which has been for the most part relying on cross-sectional designs. The purpose of the current investigation was to examine the change over time in the interplay between perceptions of the motivational climate, motivation regulations, and emotional states in competitive athletes. Specifically, we used a 3-month, two-wave repeated measures design to examine the relationship between athletes’ perceptions of the task- and ego-involving features of the motivational climate, autonomous and controlled motivations, and functional/dysfunctional and pleasant/unpleasant emotional states. A second aim of the study was to determine the temporal ordering of athletes’ emotions on motivation regulations. We tested the following four hypotheses: (a) H_0_: athletes’ motivation regulations do not predict changes in emotions, and emotions do not predict changes in motivation regulations; (b) H_1_: emotions predict changes in motivation regulations—pleasant emotions positively predict autonomous motivation and negatively predict controlled motivation, whereas unpleasant emotions positively predict controlled motivation and negatively predict autonomous motivation; (c) H_2_: motivation regulations predict changes in emotions—autonomous motivation positively predicts pleasant emotions and negatively predicts unpleasant emotions, whereas controlled motivation positively predicts unpleasant emotions and negatively predicts pleasant emotions; and (d) H_3_: emotions and motivation regulations have a reciprocal relationship—motivation regulation predicts changes in emotions, and emotions predict changes in motivation regulations.

## Materials and Methods

### Participants

The participants were 217 Finnish athletes (126 men, 91 women, *M*_age_ = 21.24 year, *SD* = 4.53). One hundred and sixty-one competed in team sports (e.g., ice hockey, soccer, floorball, and volleyball), and 56 in individual sports (e.g., swimming, karate, and track and field). One hundred and twenty-five were national level competitors and 92 were international level athletes having achieved good results in European or World Championships. The participants’ mean sport experience was 10.53 years (*SD* = 3.84), and they had trained an average of 14.15 h per week (*SD* = 5.05). Approximately two-thirds (60.45%, *n* = 359) of time 1 participants also responded to the questionnaire at time 2.

### Measures

#### Motivational Climate

A Finnish version of the Perceived Motivational Climate in Sport Questionnaire-2 (PMCSQ-2; Newton et al., 2000) was used to measure athletes’ perceptions of their motivational climate in terms of task- and ego-involving. Task-involving climate items (e.g., “the focus is to improve each game/practice”) reflect perceptions that the athlete has an important role on the team, and that co-operative learning and effort/improvement are encouraged. Ego-involving items (e.g., “players/athletes are afraid to make mistakes”) reflect feelings of intra-team rivalry among players/athletes on the team, perceptions that mistakes are punished, and that coach recognition is reserved for the most talented athletes. Each item was rated on a 5-point Likert scale ranging from 1 (*strongly disagree*) to 5 (*strongly agree*). The Finnish version of the PMCSQ-2 revealed acceptable internal consistency as administered to 494 athletes (283 men, 211 women) with α = 0.87 for both task-involving and ego-involving climates (Ruiz et al., 2017a).

#### Motivation Regulations

A Finnish version of the Behavior Regulation in Sport Questionnaire (BRSQ; Lonsdale et al., 2008) was used to assess athletes’ motivation regulations. The BRSQ comprises six 4-item subscales that measure intrinsic motivation (e.g., “because I enjoy it”), integrated regulation (e.g., “because it’s a part of who I am”), identified regulation (e.g., “because the benefits of sport are important to me”), introjected regulation (e.g., “because I would feel ashamed if I quit”), external regulation (e.g., “because people push me to play”) and amotivation (e.g., “but I question why I continue”). Each item was assessed on a 7-point Likert type scale ranging from 1 (*not at all true*) to 7 (*very true*). In this study, mean scores were calculated for autonomous and controlled styles of motivation. Adequate internal reliability of the BRSQ has been reported with α = 0.87 for autonomous motivation, α = 0.90 for controlled motivation, and α = 0.78 for amotivation (Ruiz et al., 2017a).

#### Emotional Experiences

Eight emotional modality items from psychobiosocial states scales (Robazza et al., 2016; Ruiz et al., 2016, 2018) were used to assess athletes’ emotional experiences. Each item includes 3–4 descriptors per row and is categorized as functionally helpful or harmful for performance with two items assessing: (a) functional pleasant states (“enthusiastic, confident, carefree, joyful”), and (b) functional anger (“fighting spirit, fierce, aggressive”); and two items measuring: (c) dysfunctional anxiety (“worried, apprehensive, concerned, troubled”); and (d) dysfunctional anger (“furious, resentful, irritated, annoyed”). First, athletes were asked to select one word answering the question “how do you feel right now in relation to your forthcoming performance?” Second, they rated the intensity on a scale ranging from 0 (*nothing at all*) to 4 (*very much*). Third, athletes rated the anticipated or perceived functional impact on performance on a scale ranging from +3 (*very helpful*) to −3 (*very harmful*).

### Procedure

Following approval from the local university ethics committee, data collection occurred at two time points during a 3-month period. The participants were recruited via training centers, sport schools, and clubs in five cities in Northern, Central, and Southern parts of Finland. Written consent was obtained from all participants after having explained them the purpose of the study, emphasized voluntary participation, and assured confidentiality of the results. Athletes under 18 gave their assent and a guardian provided written consent. The questionnaires were administered either individually or in small groups, in a quiet place, close to the participants training facilities. To ensure that participants had experience and awareness of the motivational aspects of the coach-created environment, data collection took place a few weeks after the beginning of the season, 30 min prior to a practice session. Participants responded to the questionnaires at time 1 (T1) and 3 months later (T2). Questionnaire administration took approximately 30 min.

### Data Analysis

**FIGURE 1:**
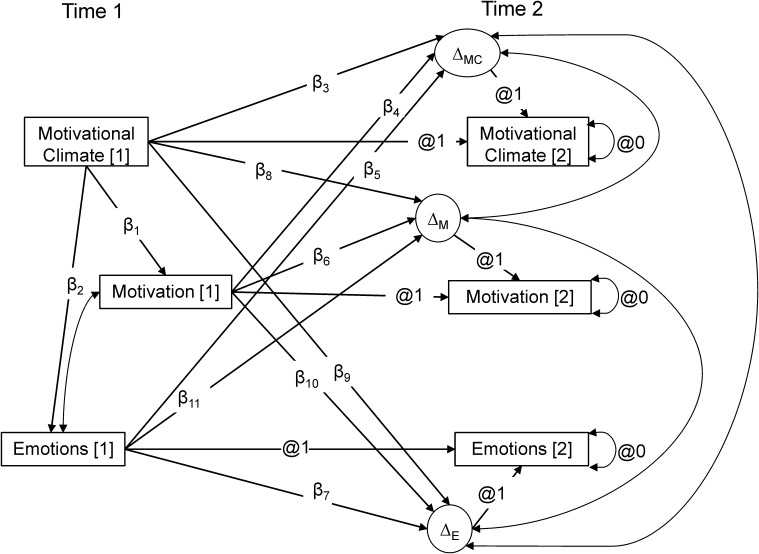
Hypothesized latent change score model of motivational climate, motivation regulations, and emotions. Δ represents latent change score.

Prior to conducting the main analysis, data were screened for inputting errors, distribution, and multivariate outliers (Tabachnick and Fidell, 2013). Twelve participants were identified as outliers (Mahalanobis distance larger than χ^2^[18] = 51.179) and were removed from further analyses. Intra-class correlations (ICC) were calculated to examine the need to conduct multilevel analysis. Statistically significant ICC were only found for task-involving climate, thus, single level analyses were conducted. Descriptive statistics, variable intercorrelations and Cronbach’s α coefficients were calculated. Structural equation modeling was conducted with Mplus 8.2 (Muthén and Muthén, 2017) using the missing-data function and adjusting for non-normality with the robust full information maximum likelihood estimator. Confirmatory factor analysis was conducted for the full measurement model at both T1 and T2. As the main analysis, latent change score modeling was used to examine the relationship between perceptions of the motivational climate, motivation relations, and athlete’s emotions (intensity and functional impact). Latent change score modeling, also called latent difference score modeling, is conducted within the framework of structural equation modeling that combines features from cross-lagged regression modeling and latent growth curves (Ferrer and McArdle, 2003; McArdle and Prindle, 2008; McArdle, 2009). In latent change score model the focus is on describing a variable *Y* at a time *t* defining ΔY*_t_* as the change in *Y* from *t – 1* to *t* (McArdle, 2009). The coefficients relating Y*_t_* and Y*_t−1_* are constrained to 1 and there is no error terms in the equation for Y*_t_*, thus Y*_t_* is directly the sum of Y*_t −1_* and ΔY*_t_*, whereΔY*_t_* can be used as a latent variable. Latent difference scores were calculated separately for task-involving climate, autonomous motivation regulations, and reported intensity and impact of each of the following emotions: functional pleasant states, anxiety, functional and dysfunctional anger. On the other hand, latent difference scores were calculated for ego-involving climate, controlled motivation regulations, and the intensity and reported impact of anxiety, functional anger, and dysfunctional anger (see Figure 1).

The fit of the path models was evaluated considering the comparative fit index (CFI), the Tucker-Lewis Index (TLI), the standardized root mean square residual (SRMR), and the root mean square error of approximation (RMSEA). As recommended by Hu and Bentler (1999), a good model fit is inferred when values of CFI and TLI are close to 0.95, the SRMR is close to 0.08, and the RMSEA is close to 0.06. The null hypothesis (H_0_) would be supported if the regression coefficients β_10_ or β_11_ (see Figure 1) were non-significantly different from zero. If coefficient β_10_, but not coefficient β_11_, was significant, H_1_ (motivational regulations predict changes in emotions) would be supported. If coefficient β_11_, but not coefficient β_10_, was significant, then H_2_ (emotions predict changes in motivational regulations) would be supported. Finally, if both coefficients β_10_ and β_11_ were significant, then H_3_ (reciprocal effects) would be supported. Effect sizes were interpreted following Cohen (1988) guidelines, whereby values of 0.2 are considered small, 0.5 are moderate, and 0.8 are large.

## Results

### Descriptive Statistics and Correlations

The athletes reported moderate to high values for perceptions of a task-involving climate, autonomous motivation, intensity and perceived impact of functional pleasant states, and low values for ego-involving climate, controlled motivation, intensity and perceived impact of dysfunctional anger, and dysfunctional anxiety at both time points (Table 1). Cronbach’s α coefficients and ω values for the scales were acceptable (all α > 0.868, and ω > 0.882) deeming the scales reliable (McNeish, 2018).

**Table 1:** Descriptive statistics and Cronbach’s alphas (α) and composite reliability (ω) of study variables.

	T1			T2		
Variable (range)	*M*	*SD*	α/ω	*M*	*SD*	α/ω
(1) Task climate (1–5)	4.01	0.50	0.889 /0.892	3.93	0.57	0.925 /0.925
(2) Ego climate (1–5)	2.58	0.61	0.880 /0.884	2.70	0.63	0.892 /0.899
(3) Autonomous motivation (1–7)	5.55	0.76	0.868 /0.882	5.55	0.80	0.888 /0.896
(4) Controlled motivation (1–7)	2.07	0.99	0.883 /0.885	2.20	1.03	0.883 /0.884
(5) Pleasant^+intensity^ (0–4)	2.55	0.69	^∗^	2.51	0.86	^∗^
(6) Anxiety^−intensity^ (0–4)	1.03	0.98	^∗^	1.10	1.12	^∗^
(7) Anger^+intensity^ (0–4)	1.74	1.09	^∗^	1.78	1.07	^∗^
(8) Anger^−intensity^ (0–4)	0.86	1.02	^∗^	1.04	1.10	^∗^
(9) Pleasant^+impact^ (−3/+3)	1.70	1.29	^∗^	1.72	1.37	^∗^
(10) Anxiety^−impact^ (−3/+3)	−1.36	1.17	^∗^	−1.51	1.19	^∗^
(11) Anger^+impact^ (−3/+3)	2.03	1.21	^∗^	1.98	1.23	^∗^
(12) Anger^−impact^ (−3/+3)	−0.83	1.29	^∗^	−0.90	1.40	^∗^

*N = 205. T1 = Time 1; T2 = Time 2 (three months later). ^∗^Individual items.*

As shown in Table 2, and following Zhu (2012) criteria, positive low correlations were observed between perceptions of a task-involving climate and autonomous motivation, and between perceptions of an ego-involving climate and controlled motivation at both times. Positive low correlations were also observed between task-involving climate and the perceived impact of functional anger, while an ego-involving climate was positively correlated with the intensity and perceived impact of dysfunctional anger. Weak or no correlations were found between the intensity and the perceived impact of athletes’ emotions.

**Table 2:** Bivariate correlations of the study variables in Time 1 and Time 2.

	1	2	3	4	5	6	7	8	9	10	11	12	13	14	15	16	17	18	19	20	21	22	23
*Time 1*																							
(1) TC																							
(2) EC	−0.37																						
(3) AM	0.36	−0.12																					
(4) CM	−0.07	0.23	0.03																				
(5) P^+intensity^	0.08	−0.08	0.19	−0.03																			
(6) Ax^−^-^intensity^	−0.21	0.19	−0.01	0.17	−0.15																		
(7) Ag^+intensity^	0.07	0.12	0.18	−0.12	0.19	0.18																	
(8) Ag^−^-^intensity^	−0.22	0.21	−0.09	0.17	−0.14	0.46	0.14																
(9) P^+impact^	0.11	−0.08	0.23	−0.12	0.11	0.01	0.23	−0.07															
(10) Ax^−^–^impact^	−0.06	0.08	−0.17	−0.11	0.01	−0.01	0.07	−0.05	−0.07														
(11) Ag^+impact^	0.28	−0.07	0.20	−0.07	0.06	−0.03	0.24	−0.05	0.41	−0.10													
(12) Ag^−impact^	−0.14	0.14	−0.08	−0.02	0.02	−0.09	−0.13	0.00	−0.05	0.34	−0.09												
*Time 2*																							
(13) TC	0.68	−0.29	0.26	−0.03	0.11	−0.05	0.07	−0.14	0.16	−0.07	0.30	−0.16											
(14) EC	−0.30	0.69	−0.09	0.18	−0.02	0.18	0.10	0.25	−0.07	0.13	−0.19	0.07	−0.44										
(15) AM	0.39	−0.14	0.75	0.03	0.13	0.00	0.14	−0.01	0.15	−0.18	0.20	−0.12	0.34	−0.13									
(16) CM	−0.18	0.26	0.05	0.76	−0.06	0.26	−0.11	0.22	−0.03	−0.06	−0.11	0.12	−0.16	0.29	−0.05								
(17) P^+intensity^	0.06	−0.08	0.04	0.13	0.23	−0.04	0.03	0.04	0.15	−0.11	0.14	−0.08	0.10	−0.08	0.21	0.12							
(18) Ax^−intensity^	−0.19	0.12	−0.01	0.21	−0.02	0.27	−0.02	0.18	0.06	−0.14	−0.08	0.08	−0.19	0.20	−0.02	0.29	−0.17						
(19) Ag^+intensity^	−0.02	0.03	0.01	0.11	0.16	0.09	0.30	0.15	0.13	−0.02	0.06	0.07	−0.10	0.17	0.02	0.14	0.31	0.04					
(20) Ag^−intensity^	−0.17	0.22	0.03	0.09	0.00	0.25	0.06	0.29	−0.04	0.04	−0.12	0.07	−0.19	0.32	0.00	0.24	−0.12	0.45	0.10				
(21) P^+impact^	0.11	−0.20	0.16	0.04	0.01	0.06	−0.03	−0.01	0.21	−0.16	0.16	−0.13	0.15	−0.16	0.08	0.08	0.34	−0.11	0.21	−0.09			
(22) Ax^−impact^	−0.17	0.18	−0.18	0.08	0.01	−0.02	−0.11	0.08	−0.18	0.39	−0.22	0.26	−0.09	0.12	−0.22	0.12	−0.12	0.02	−0.11	0.09	−0.10		
(23) Ag^+impact^	0.14	−0.07	0.12	0.05	0.05	0.01	0.10	0.06	0.28	−0.16	0.35	−0.05	0.12	−0.05	0.16	−0.04	0.17	−0.08	0.32	−0.13	0.25	−0.21	
(24) Ag^−impact^	−0.16	0.24	0.03	0.10	0.00	0.02	−0.04	0.05	−0.03	0.26	0.02	0.50	−0.18	0.14	−0.10	0.18	−0.17	0.01	−0.08	0.05	−0.15	0.22	−0.06

*Bivariate correlations of 0.13 and above are significant at p < 0.05; bivariate correlations of 0.18 and above are significant at p < 0.01; TC, task-involving climate; EC, ego-involving climate; AM, autonomous motivation; CM, controlled motivation; P, pleasant states; Ag, anger; Ax, anxiety; +, functional; −, dysfunctional.*

### Confirmatory Factor Analysis

Following recommendations by Little et al. (2002) and to improve the ratio of variable to sample size, we created construct-specific parcels. Specifically, six parcels were created following the theoretical structure of motivational climate (Newton et al., 2000). Three parcels were defined for task-involving climate by calculating the sums of the items representing the second-order dimensions of cooperative learning, important role, and effort/improvement. The remaining items representing punishment for mistakes, unequal recognition, and intra-team member rivalry were assigned to the three parcels defined for ego-involving climate. In line with SDT (Deci and Ryan, 1985) conceptualization and Ryan and Connell (1989) suggestion, four parcels were defined for autonomous motivation by calculating the sums of items representing intrinsic motivation, integrated regulation, and identified regulation. The remaining items representing introjected regulation and external regulation were allocated to four parcels for controlled motivation. Amotivation was excluded from the analysis because we were interested in the quality of motivation rather than the quantity of motivation. Overall, acceptable model fit was obtained for the full measurement model representing perceived motivational climate, motivation regulations, functional emotions, and dysfunctional emotions at T1, χ^2^/df = 8.436, RMSEA = 0.057, CFI = 0.924, TLI = 0.909, SRMR = 0.065, and at T2, χ^2^/df = 8.7653, RMSEA = 0.043, CFI = 0.958, TLI = 0.950, SRMR = 0.068.

### Structural Equation Modeling

A total of 14 structural models were tested to examine the temporal ordering of motivation regulations and the intensity and perceived impact of emotions. Specifically, four models were estimated including paths relating task-involving climate and autonomous motivation with the intensity of functional pleasant states, dysfunctional anxiety, functional anger, and dysfunctional anger separately (Models 1–4). Three other models included paths relating ego-involving climate and controlled motivation with the intensity of dysfunctional anxiety, functional anger, and dysfunctional anger (Models 5–7). Similarly, seven models were tested to examine the relationships with impact ratings of emotions (Models 8–14). All models were saturated. In regards to emotion intensity, one additional path was included in the model from dysfunctional anger intensity to the latent difference score of ego-involving climate (M7). We also added a path going from functional anger impact ratings to the latent difference score of ego-involving climate (M13).

Overall, a task-involving climate was a positive predictor of autonomous motivation at T1 (see Table 3, M1–M4 and M8–M11, β_1_) and of the latent change in autonomous motivation at T2 (M1–M4 and M8–M11, β_8_). A task-involving climate was a negative predictor of the intensity of anxiety and dysfunctional anger at T1 (M2-β_2_ and M4-β_2_), and the latent change in these emotions at T2 (M2-β_9_ and M4-β_9_), while it positively predicted the reported impact of functional anger at T1 (M10-β_2_), but not the change in this emotion at T2 (M10-β_9_). An ego-involving climate positively predicted controlled motivation at T1 (M5–M7 and M12–M14, β_1_) and the latent difference in controlled motivation at T2, but only for the path including the intensity of functional anger (M6-β_8_). Ego-involving climate was a positive predictor of the intensity of anxiety at T1 (M5-β_2_), the intensity and reported impact of dysfunctional anger at T1 (M7-β_2_ and M14-β_2_, respectively), and latent change in the intensity and reported impact of dysfunctional anger at T2 (M7-β_9_ and M14-β_9_, respectively). Effect sizes for these reported significant paths were low.

**Table 3:** Standardized path coefficients for relationships between motivational climate (MC), motivation regulations (M), and emotions (E).

			MC_1_-M_1_	MC_1_-E_1_	MC_1_-ΔMC	M_1_-ΔMC	E_1_-ΔMC	M_1_-ΔM	E_1_-ΔE	MC_1_-ΔM	MC_1_-ΔE	M_1_-ΔE	E_1_-ΔM
Model			(β_1_)	(β_2_)	(β_3_)	(β_4_)	(β_5_)	(β_6_)	(β_7_)	(β_8_)	(β_9_)	(β_10_)	(β_11_)
M1 TC	AM	P^+int^	0.36^∗∗∗^	0.08	−0.27^∗∗∗^	0.02	0.07	−0.35^∗∗∗^	−0.51^∗∗∗^	0.21^∗∗^	0.05	−0.02	−0.03
M2 TC	AM	Ax^−int^	0.36^∗∗∗^	−0.21^∗∗^	−0.24^∗∗∗^	0.02	0.13	−0.35^∗∗∗^	−0.56^∗∗∗^	0.22^∗∗^	−0.13^∗^	0.04	0.05
M3 TC	AM	Ag^+int^	0.36^∗∗∗^	0.07	−0.27^∗∗∗^	0.03	0.03	−0.35^∗∗∗^	−0.59^∗∗∗^	0.21^∗∗^	−0.02	−0.03	0.00
M4 TC	AM	Ag^−int^	0.36^∗∗∗^	−0.22^∗∗^	−0.27^∗∗∗^	0.03	0.02	−0.35^∗∗∗^	−0.57^∗∗∗^	0.23^∗∗^	−0.13^∗^	0.10	0.13^∗^
M5 EC	CM	Ax^−int^	0.23^∗∗∗^	0.19^∗∗^	−0.37^∗∗∗^	0.02	0.06	−0.34^∗∗∗^	−0.56^∗∗∗^	0.10	0.03	0.14^∗^	0.18^∗^
M6 EC	CM	Ag^+int^	0.23^∗∗∗^	0.12	−0.37^∗∗∗^	0.03	0.03	−0.33^∗∗∗^	−0.59^∗∗∗^	0.14^∗^	−0.04	0.13^∗^	−0.05
M7 EC	CM	Ag^−int^	0.23^∗∗∗^	0.21^∗∗^	−0.39^∗∗∗^	0.00	0.14	−0.33^∗∗∗^	−0.58^∗∗∗^	0.11	0.14^∗^	0.01	0.12
M8 TC	AM	P^+pi^	0.36^∗∗∗^	0.11	−0.27^∗∗∗^	0.01	0.11	−0.34^∗∗∗^	−0.63^∗∗∗^	0.21^∗∗^	0.05	0.08	−0.06
M9 TC	AM	Ax^−pi^	0.36^∗∗∗^	−0.06	−0.27^∗∗∗^	0.02	0.04	−0.36^∗∗∗^	−0.55^∗∗∗^	0.21^∗∗^	−0.12	−0.07	−0.08
M10 TC	AM	Ag^+pi^	0.36^∗∗∗^	0.28^∗∗∗^	−0.31^∗∗∗^	0.01	0.16	−0.36^∗∗∗^	−0.58^∗∗∗^	0.20^∗∗^	0.03	0.03	0.04
M11 TC	AM	Ag^−pi^	0.36^∗∗∗^	−0.14	−0.28^∗∗∗^	0.03	−0.09	−0.35^∗∗∗^	−0.45^∗∗∗^	0.20^∗∗^	−0.14	0.12	−0.07
M12 EC	CM	Ax^−pi^	0.23^∗∗∗^	0.08	−0.37^∗∗∗^	0.04	0.09	−0.32^∗∗∗^	−0.54^∗∗∗^	0.13	0.12	0.08	0.02
M13 EC	CM	Ag^+pi^	0.23^∗∗∗^	−0.07	−0.37^∗∗∗^	0.01	0.18	−0.32^∗∗∗^	−0.56^∗∗∗^	0.13	−0.06	0.08	−0.08
M14 EC	CM	Ag^−pi^	0.23^∗∗∗^	0.14^∗^	−0.36^∗∗∗^	0.02	−0.03	−0.31^∗∗∗^	−0.46^∗∗∗^	0.10	0.16^∗∗^	0.08	0.20^∗∗^

*Δ, latent change score between Time 1 and Time 2 (three months later). TC, task-involving climate; EC, ego-involving climate; AM, autonomous motivation; CM, controlled motivation; P, pleasant states; Ag, anger; Ax, anxiety; +, functional; −, dysfunctional; int, intensity; pi, perceived impact. ^∗^p < 0.05; ^∗∗^p < 0.01; ^∗∗∗^p < 0.001.*

As can be observed in Table 3, the path from the reported intensity of dysfunctional anger at T1 to the latent change in autonomous motivation at T2 (M4-β_11_) was significant and positive, but the coefficient from autonomous motivation at T1 to the latent change in dysfunctional anger at T2 (M4-β_10_) was non-significant. Also, the path from the reported impact of dysfunctional anger at T1 to the latent change in controlled motivation (M14-β_11_) was significant and positive, but β_10_ was non-significant. These findings regarding the intensity and perceived impact of dysfunctional anger would support H_1_ (emotion predicts changes in motivational regulations). The path from controlled motivation at T1 to the latent change in the intensity of functional anger at T2 (M6-β_10_) was significant, but not β_11_, supporting H_2_ (motivational regulations predict changes in emotions). Finally, the path from controlled motivation at T1 to the latent change in reported intensity of anxiety at T2 (M5-β_10_) and the path from the intensity of anxiety at T1 to the latent change in controlled motivation at T2 (M5-β_11_) were significant and positive, thus providing support for H_3_ (reciprocal effects). Effect sizes for these reported significant paths were also low.

## Discussion

The current study examined the relationship between athletes’ perceptions of their motivational climate, motivation regulations, intensity, and reported functional impact of pleasant and stress-related emotions over time. We expected that perceptions of task-involving climate would positively predict athletes’ autonomous motivation and functional emotions, and that this relationship would hold over time. In contrast, an ego-involving climate was expected to be a positive predictor of controlled motivation and dysfunctional emotions. A main aim of this study involved testing the temporal ordering of athletes’ emotions and motivation regulations by investigating two different sequences. The first sequence examined the mediating role of motivation regulations in the motivational climate and emotion relationships, while the second sequence placed emotions as mediators of the motivational climate and motivation regulations relationship.

The results indicated moderately high positive correlations in the reported scores of perceived motivational climate across time. These findings concur with empirical evidence from a previous longitudinal study examining the perceptions of football players about their motivational climate at the beginning and at the end of the season (Sage and Kavussanu, 2008). A slightly lower stability was found in the Sage and Kavussanu’s study. However, their study included other variables (i.e., goal orientations and moral behaviors), which may have suppressed the magnitude of the values. In addition, the timeframe in their study was relatively longer including data from the beginning to the end of the season, which may also have allowed for other aspects (e.g., performance outcome) to influence the perceptions of the motivational climate. Moderately high positive correlations were also found in the participants’ reported motivation regulations. This finding is in line with the Lonsdale and Hodge (2011) study results on elite level athletes assessed over a 4-month period. However, in regards to athletes’ emotional states, low positive correlations were found for the emotion intensity. These results concur with Hanin (2000) assumption about intra-individual variability of emotion intensity as well as with Lazarus (2000) conceptualization of emotions as individuals’ responses to a transaction with the environment that unfolds over time. Empirical support for variability in emotional intensity has derived from studies assessing the intensity of anxiety 1 h prior to four meets (Turner and Raglin, 1996) or a range of feeling states 15 min prior a fight in 10 competitions across the entire season (Robazza et al., 2004). In regards to the functional impact, positive low correlations were reported in the case of stress-related emotions, with the exception of dysfunctional anger where a moderate correlation was found across times (*r* = 0.50). These results indicate that meta-experiences reflecting athletes’ awareness of the impact, preferences, or attitudes toward emotions (Hanin, 2004) are more stable than emotional states.

As expected, significant positive paths were found from the perceptions of a task-involving climate to the changes in athletes’ autonomous motivation (Table 3), although effect size was low. However, partial support was obtained for the hypothesized link between ego-involving climate and the change in controlled motivation, as only one significant positive path was found with the intensity of functional anger included in the model, but not in the case of other emotions. Negative significant paths were found between task-involving climate and the change in intensity of dysfunctional anxiety and dysfunctional anger. In contrast, significant positive paths were found for ego-involving climate and the change in intensity and reported impact of dysfunctional anger. Notably, small effect sizes of significant paths were observed. These findings indicate that the perceptions of a motivational climate have a carryover effect on athletes’ emotional experiences, especially on anger and anxiety. The results are in line with AGT (Nicholls, 1989) and SDT (Deci and Ryan, 1985, 2000; Ryan and Deci, 2017) assumptions that a task-involving climate is associated with a more adaptive achievement pattern while an ego-involving climate is associated with a more maladaptive pattern. Taken together, these results confirm our hypothesis regarding the stability of the interplay between motivational climate, motivation, and emotions supporting the notion that the social situation created by significant others influences goal involvement and how participants interpret their experiences.

Our findings showed that the relationship between athletes’ quality of motivation and emotions varied depending on the type of motivation and emotions assessed. Specifically, the first hypothesis (emotions predict changes in motivation regulations) was supported by the significant links found between the intensity of dysfunctional anger and the change score in autonomous motivation, and between dysfunctional perceptions of anger and the change score for controlled motivation. The links in the opposite directions were non-significant. The second hypothesis (motivation regulations predict changes in emotions) was partially supported by a significant path from controlled motivation to the change score in intensity of functional anger, while a non-significant link was found in the opposite direction. The third hypothesis (reciprocal relationship between emotions and motivation regulations) was supported by significant paths between controlled motivation and the change score of the intensity of anxiety in both directions. Taken together, the results suggest that the interplay between motivation and emotions is contingent of the specific emotions. Different findings were observed regarding the intensity and functional impact of emotions. Thus, the findings also provide support for the assessment of both intensity and functional impact of emotions. However, effect sizes were low, thus, overall findings should be interpreted with caution.

The notion that motivation determines emotions is supported by several theorists. For instance, in addition to AGT (Nicholls, 1989) and SDT (Deci and Ryan, 2000; Ryan and Deci, 2017), in the contextual motivation sequence proposed within the hierarchical model (Vallerand, 1997) it is assumed that motivation determines behavior, emotions, and thoughts. Also Lazarus (2000) cognitive-motivational-relational theory of emotion conceptualizes emotion as an organized psychophysiological reaction reflecting person-environment relationships. Emotions are the results of an individual’s appraisal of a situation in terms of goal relevance and congruence. According to Lazarus, the function of emotions is to facilitate adaptation. Similarly, the IZOF model (Hanin, 2000, 2007) assumes that emotions are triggered by person’s appraisals of the probability of achieving relevant goals, and the interaction of both functional and dysfunctional emotions influences performance.

Previous research, with young participants in particular, has also examined emotions as both antecedents and consequences of motivation. For instance, Blanchard et al. (2009) found that self-determined motivation predicted higher intensity levels of positive emotions in young basketball players. In addition, intervention studies have provided evidence for the influence of motivation in the individuals’ pleasant emotional experiences such as enjoyment (Smith et al., 2007; MacPhail et al., 2008). In contrast, in the Bortoli et al. (2014) study, pleasant and unpleasant states were included as mediators in the relationship between motivational climate and motivation regulations.

Our results indicate that only in the instance of controlled motivation and the intensity of anxiety there were significant paths indicating a reciprocal relationship. This may support the notion that emotions and motivation are complex phenomena. Lazarus (1999) suggested using a systems theory approach whereby each subsystem would be comprised of several variables, and thus, it would be possible to assume that sometimes one may act as an independent variable and at other times as an outcome variable. According to a systems theory approach, in the IZOF model emotions are conceptualized as core components of a psychobiosocial state, which can be manifested in several interrelated modalities including emotional and motivational aspects (for descriptions, see Hanin, 2010; Ruiz et al., 2016). Based on our results, significant emotion-motivation relationships emerged on the reported data on anxiety and anger, but not on pleasant experiences.

### Strengths, Limitations, and Future Research Directions

This study was one of the first to explore the sequential interplay between the quality of motivation and performance related emotions in sport. The repeated measures design allowed the examination of two alternative sequences in which motivational climate would serve as antecedents of: (1) the variability in motivation regulations, which would result in different emotions; or (2) different emotions, which would be antecedents of the quality of motivation. Overall, results indicate that emotions and motivation are intertwined: specific emotions predicted different types of motivation and, at the same time, motivation regulations predicted specific emotions.

The study has some limitations that should to be addressed in the future. First, because of the relatively small sample size, we estimated the models for each emotion separately. Previous studies have used a composite index for motivation (Lonsdale and Hodge, 2011). However, because emotions reflect different meanings we opted for separate analysis on the relationship between autonomous/controlled motivation and different emotions. Future research should attempt to replicate the present findings with a larger sample to allow for the estimation of a model including all study variables. Effect sizes obtained in the study were relatively small, thus, larger sample studies are warranted in the replication of these findings. Second, we used repeated measures at two time points across 3 months. This allowed us to examine inter-individual variability in intra-individual patterns of change over time. However, future research could include a larger number of measurement points, which would provide a more reliable assessment and information about individual trajectories, thereby shedding more light into the understanding of the interplay of motivational and emotional variables across time. A final limitation of the study is the use of a correlational design. Thus, future experimental research where some of the studied variables are manipulated would allow for a direct test of the proposed models, providing a better understanding of the nature of the motivation and emotion relationship. Another important avenue for future research would be the examination of the role of athletes’ basic psychological needs satisfaction as potential mediators in this relationship.

### Practical Implications

The study has important practical implications. Findings support the notion that coaches need to promote a task-involving motivational climate to attain long lasting positive effects on autonomous motivation. They also need to decrease dysfunctional anxiety and anger to enhance the athletes’ adaptation to psychological stress associated with performance in high achievement contexts. Coaches should also be mindful that an ego-involving climate could have negative long-term effects by triggering controlled motivation and dysfunctional stress-related emotions. Sport psychology practitioners should help athletes become aware of the personal reasons to participate in sport, their emotional experiences, and the interplay between motivation and emotions. Sport psychology interventions could focus on an early identification of athletes presenting high levels of controlled motivation or dysfunctional anxiety, in order to prevent maladaptive responses to psychological stress and their negative long-term effects.

## Data Availability

The datasets generated for this study are available on request to the corresponding author.

## Ethics Statement

This study was carried out in accordance with ‘the ethical principles of research in the humanities and social and behavioral sciences and proposals for ethical review’ drafted by the National Advisory Board on Research Ethics in Finland (TENK). The University of Jyväskylä Ethical Committee approved the research protocol. In accordance with the Declaration of Helsinki, all participants gave written informed consent, after anonymity and confidentiality was assured.

## Author Contributions

All authors listed have made a substantial, direct and intellectual contribution to the work, and approved it for publication.

## Conflict of Interest Statement

The authors declare that the research was conducted in the absence of any commercial or financial relationships that could be construed as a potential conflict of interest.
